# Supplementary material for: Epidemiology, Diagnostic Strategies, and Therapeutic Advances in Diffuse Midline Glioma
Source: J Clin Med. 2023 Aug 12;12(16):5261. doi: 10.3390/jcm12165261 (PMC10456112; doi:10.3390/jcm12165261)
Supplement: Supplementary file 1 [file jcm-12-05261-s001.zip › jcm-2531206-supplementary.pdf]

Supplementary Materials

Table S1. Highlights of results of clinical studies.

Author/ year	Objectives	n	Treatment regimen used	Results	Level of evidence
**Bailey et al 2013 [1]**	To assess the response to treatment of DIPG treated with prolonged temozolomide and radiotherapy.	43	RT and concomitant temozolomide (75 mg/m2) then up to 12 21-day cycles of adjuvant temozolomide (75-100 mg/m2)	No improved survival could be demonstrated with the addition of dense doses of temozolomide to standard radiotherapy in children with DIPG.	B2b
**Pai Panandiker et al 2014 [2]**	To analyse the effect of time from diagnosis to initiation of radiotherapy in children with DIPG.	95	RT was administered in doses of 54.0 to 55.8 Gy at 1.8 Gy per fraction.	No significant benefit from reducing the time between diagnosis and initiation of radiotherapy was demonstrated. Although the authors suggest that early initiation of therapy is desirable, the findings do not support intensive efforts to shorten delays.	B3b
**Jakacki et al 2016 [3]**	Determine whether lomustine and temozolomide following radiotherapy and concurrent temozolomide on DIPG	108	To determine whether lomustine and temozolomide after concurrent radiotherapy and temozolomide in DIPG	There was significant improvement in symptoms and during follow-up when combining TMZ	C4
**Kebudi et al 2019 [4]**	Describe nimotuzumab-containing regimens for the treatment of DIPG	24	Nimotuzumab: 150 mg/m2 1/week for 12 weeks, then every two weeks with TMZ until progressive disease. Patients with progressive disease were added vinorelbine until death.	Nimotuzumab-containing regimens are feasible and tolerable; it may be that some patients with newly diagnosed DIPG or progressive disease may benefit modestly. No improved two-year survival was demonstrated compared to other	C4

				investigations with large cohorts.	
**Baxter et al 2020 [5]**	To evaluate the use of veliparib (ABT-888) with radiation and temozolomide in children with DIPG.	65	Veliparib: Monday to Friday BID + radiotherapy, then 4 weeks rest, finally veliparib at 25 mg/m2 BID and TMZ 135 mg/m2 OD for 5 days every 28 days.	The drug was tolerated but did not improve survival.	A1b
**Mueller et al 2020 [6]**	To evaluate the safety and efficacy of a peptide vaccine targeting H3.3K27M. stratum A (DIPG) or stratum B (non-pontine MGD).	19	Vaccine + poly-ICLC every 3 weeks for 8 cycles, followed by once every 6 weeks.	The H3.3K27M-specific vaccine was well tolerated, H3.3K27M-specific CD8+ immune responses developed, and in patients in whom it was administered and immune response was achieved, overall survival was longer compared to non-responders.	C4
**Gállego Pérez-Larraya et al 2022 [7]**	Evaluating the use of a DNX-2401 oncolytic virus for DIPG	12	1×10^10 (the first 4 patients) or 5×10^10 (the next 8 patients) DNX-2401 viral particles, and 11 received subsequent radiotherapy.	Intratumoural infusion of DNX-2401 prior to radiotherapy is feasible in children with DIPG, was successful in causing changes in T-cell activity and a reduction or stabilisation of tumour size in some patients, however, further studies are required to assess safety and adverse effects.	C4
**Gardner et al 2022 [8]**	To evaluate the dose escalation and expansion of the single agent ONC201 in pediatric patients.	22	Standard dose escalation therapy of 3 + 3. The target dose was the previously established RP2D for adults (625 mg), scaled by body weight.	The adult RP2D of 625 mg ONC201 weekly escalated by body weight was well tolerated. Further investigation of ONC201 for DIPG/DMG is justified.	C4
**Majzner et al 2022 [9]**	Evaluating GD2-CAR T-	-	CTX is lymphocyte depletion	The findings confirmed that GD2	A1b

	cell therapy for H3K27M-mutated diffuse midline gliomas		(cyclophosphamide 500 mg m2 daily and fludarabine 25 mg m 2 daily on days -4, -3 and -2), then infusion of CAR T EV cells on day 0. In some cases second infusion, CAR T ICV via Ommaya. Case: 1 fixed dose of 50 million CAR T cells after an increase in lymphocyte-depleting chemotherapy (cyclophosphamide 600 mg m 2 daily and fludarabine 30 mg m 2daily on days -5, -4, -3 and -2) All other cases: 30 million CAR T cells or their dose equivalent based on DL1 weight, whichever was lower, ICV without lymphodepletion.	can be a safe therapeutic strategy with CAR T cells.	
**Panigrahy et al 2022 [10]**	To assess whether in vivo measurements of cellular metabolites by magnetic resonance spectroscopy can serve as biomarkers of response to therapy, including progression.	46	14 cases of DIPG-associated antigenic peptide vaccines	The myoinositol/choline ratio could be useful as a prognostic biomarker after RT.	C4

Table S2. Highlights of results of preclinical studies in animals and in culture.

Author/ year	Type of study	Objectives	Results	Level of evidence
**Mount et al 2018 [11]**	*In vitro*	Describe the anti-tumour efficacy of anti-GD2 CAR T lymphocytes in H3-K27M + DIPG	Cultures of patient-derived H3-K27M mutant glioma cells exhibit high and uniform expression of the disialoganglioside GD2.	C4
**Wei et al 2018 [12]**	*In vitro*	Perform bioinformatic analysis of microarray data to reveal the pathogenesis of diffuse intrinsic pontine glioma.	This study provides new insights into the molecular mechanisms for the progression of DIPG and suggests directions for future studies.	B2b
**Bellat et al 2020 [13]**	In animal samples	Overcoming the delivery barrier through CED and maintaining a therapeutic concentration at the glioma site with a precursor of NFP	Prolonged retention of the drug at the infusion site and maintenance of an effective therapeutic effect against DIPG was achieved.	C4

Table S3. Highlights of results from review studies.

Author/ year	Objectives	Results	Level of evidence
**Grimm and Chamberlai 2013 [14]**	Review information on the DIPG	DIPG is the most common truncal tumour in children, the median age of onset is usually around 6.5 years and the median survival is less than 1 year, the standard treatment at the time of this review was fractionated radiotherapy, and the prognosis of the tumour is poor.	B3a
**Buczkowicz and Hawkins 2015 [15]**	Describe the most common epigenetic and molecular genetic signatures of DIPG in the context of molecular subgroups and histopathological diagnosis.	The most common DIPG-related mutations (ACVR1 H3F3A; K27M) are described; these authors state that histone H3 and ACVR1 mutations, the molecular subtypes of DIPG, and the rediscovered role of biopsy are redefining what is clinically possible in the treatment of this type of cancer.	B2a
**Vanan and Eisenstat 2015 [16]**	Review information on DIPG in children.	DIPGs are a heterogeneous group of tumours that are biologically distinct from other high-grade gliomas.	B3a
**González et al 2017 [17]**	Provide up-to-date information to allow a detailed understanding of DIPGs and thus help	Although the prognosis for focal gliomas has improved in recent years, for DIPGs, there are no such marked advances and the prognosis	B3a

	to optimise the management of this condition in the paediatric population.	remains bleak, however, the information available offers promising changes for better outcomes, concluding that understanding the molecular biology of the tumour is fundamental to offer therapies.	
**Gwak and Park 2017 [18]**	Synthesise the most relevant aspects of chemotherapy development for DIPG.	Convection-enhanced delivery could be used to infuse drugs directly into the brainstem parenchyma.	B3a
**Johung and Monje 2017 [19]**	Review the clinical features and current challenges of DIPG treatment, and discuss emerging insights into the unique genomic and epigenomic mechanisms driving pathogenesis.	Due to the increased availability of tissue by biopsy and rapid autopsy in preclinical research, new strategies for genomic and epigenetic classification of DIPGs have been developed.	B3a
**Lapin et al 2017 [20]**	Gathering genomic information on DIPG	Aberrations have been discovered in critical gene drivers, including histone H3, ACVR1, TP53, PDGFRA and Myc	B2a
**Hoffman et al 2018 [21]**	Compile the DIPG registries of the International and European Society of Paediatric Oncology on clinical, radiological and histomolecular features among short-term and long-term survivors.	More than 1000 tumour cases from patients in North America, Australia, Germany, Austria, Switzerland, the Netherlands, Italy, France, the UK and Croatia were analysed, reporting an overall 2-year survival of 9.6% and 1-year survival of 42.3%. The authors point out that variations in 1-year mortality could be due to factors related to cohort heterogeneity, or to accounting for cases without true DIPGs, highlighting the importance of recent molecular diagnostic strategies.	A1a
**Pollack et al 2019 [22]**	To synthesise current information on management, biological knowledge and future directions in brain tumours.	Treatment is evolving from the historical standard of conventional RT + CTX to a more nuanced and individualised approach.	B3a
**Vitanza and Monje 2019 [23]**	It provides a clinical background, through a systematic app[24]roach to diagnosis and initial care, and synthesises historical, modern and future directions for treatment.	Understanding the chromatin, signalling and immunological biology of DIPG may soon lead to clinical advances.	B3a

**Park et al 2020 [24]**	Purchasing hypofractionated versus conventional radiotherapy for diffuse intrinsic pontine glioma	Both techniques provide similar survival results for patients with DIPG.	A1a
**Argersinger et al 2021 [25]**	Discusses the controversies surrounding tumour biopsy and summarises the molecular characteristics of tumours that are therapeutic targets.	They state that what was known as DIPG has been renamed to diffuse midline glioma with H3K27M mutation, due to the specific identification of the tumour-causing mutation, which has opened the way to potential therapeutic targets. In *vitro*, animal studies and preclinical results have shown good tolerance to new therapeutic options (such as enhanced convective delivery, opening of the blood-brain barrier and immunotherapy).	B2a
**Hauser 2021 [26]**	To give an overview of the most relevant aspects of the classification and treatment of paediatric gliomas in the molecular era.	The most promising approach is based on the highly immunogenic characteristics of high-grade gliomas, which can be utilised by reactivating ineffective immune self-protective mechanisms.	B3a
**Louis et al 2021 [27]**	Summarise the WHO classification of central nervous system tumours 2021	This classification includes many mutant DIPGs as high-grade tumours, with H3K27M being the most representative. The authors specify that CNS5, like its predecessors, should be seen as a work in progress, as a stage in the evolution of CNS tumour classification.	A1a
**Damodharan et al 2022 [28]**	Review information on DIPG from general aspects and the evolving molecular landscape and treatment.	The current environment points to epigenetic modifiers and immunotherapy as the next wave of therapies along with the advancement of surgical and therapeutic barriers to bypass the blood-brain barrier.	B2a
**Liu et al 2022 [29]**	Describe how immunogenic cell death enhances DIPG immunotherapy.	The induction of immunogenic cell death was accompanied by the release of molecular patterns associated with damage.	B3a

Legend: BID: Twice daily, CART: Chimeric Antigen Receptor, CED: Convection Enhanced Delivery, CNS5: WHO Classification of Tumours of the Central Nervous System (5th version).CT: Clinical Trial, CT-A: Clinical trial on animals, CT-INV: Clinical trial on cell cultures (in vitro), CTX: Chemotherapy, DIPG: Diffuse Intrinsic Pontine Glioma, DL1: Lethal dose 1, ICV: Intracerebroventricular, NFP: Peptide nanofibre precursor, OD: Once a day, poly-ICLC: Polyinosinic acid-polycytidylic acid-poly-I-lysine carboxymethylcellulose, REV: Review, TMZ: Temozolamide.

References

1. Bailey, S.; Howman, A.; Wheatley, K.; Wherton, D.; Boota, N.; Pizer, B.; Fisher, D.; Kearns, P.; Picton, S.; Saran, F.; et al. Diffuse intrinsic pontine glioma treated with prolonged temozolomide and radiotherapy – Results of a United Kingdom phase II trial (CNS 2007 04). *Eur. J. Cancer* **2013**, *49*, 3856–3862, doi:10.1016/j.ejca.2013.08.006.
2. Panandiker, A.S.P.; Wong, J.K.; Nedelka, M.A.; Wu, S.; Gajjar, A.; Broniscer, A. Effect of time from diagnosis to start of radiotherapy on children with diffuse intrinsic pontine glioma. *Pediatr. Blood Cancer* **2014**, *61*, 1180–1183, doi:10.1002/pbc.24971.
3. Jakacki, R.I.; Cohen, K.J.; Buxton, A.; Krailo, M.D.; Burger, P.C.; Rosenblum, M.K.; Brat, D.J.; Hamilton, R.L.; Eckel, S.P.; Zhou, T.; et al. Phase 2 study of concurrent radiotherapy and temozolomide followed by temozolomide and lomustine in the treatment of children with high-grade glioma: A report of the Children's Oncology Group ACNS0423 study. *Neuro. Oncol.* **2016**, *18*, 1442–1450, doi:10.1093/neuonc/now038.
4. Kebudi, R.; Cakir, F.B.; Bay, S.B.; Gorgun, O.; Altınok, P.; Iribas, A.; Agaoglu, F.Y.; Darendeliler, E. Nimotuzumab-containing regimen for pediatric diffuse intrinsic pontine gliomas: a retrospective multicenter study and review of the literature. *Child's Nerv. Syst.* **2019**, *35*, 83–89, doi:10.1007/s00381-018-4001-9.
5. Baxter, P.A.; Su, J.M.; Onar-Thomas, A.; Billups, C.A.; Li, X.-N.; Poussaint, T.Y.; Smith, E.R.; Thompson, P.; Adesina, A.; Ansell, P.; et al. A phase I/II study of veliparib (ABT-888) with radiation and temozolomide in newly diagnosed diffuse pontine glioma: a Pediatric Brain Tumor Consortium study. *Neuro. Oncol.* **2020**, *22*, 875–885, doi:10.1093/neuonc/noaa016.
6. Mueller, S.; Taitt, J.M.; Villanueva-Meyer, J.E.; Bonner, E.R.; Nejo, T.; Lulla, R.R.; Goldman, S.; Banerjee, A.; Chi, S.N.; Whipple, N.S.; et al. Mass cytometry detects H3.3K27M-specific vaccine responses in diffuse midline glioma. *J. Clin. Invest.* **2020**, *130*, 6325–6337, doi:10.1172/JCI140378.
7. Pérez-Larraya, J.G.; Garcia-Moure, M.; Labiano, S.; Patiño-García, A.; Dobbs, J.; Gonzalez-Huarriz, M.; Zalacain, M.; Marrodan, L.; Martinez-Velez, N.; Puigdelloses, M.; et al. Oncolytic DNX-2401 Virus for Pediatric Diffuse Intrinsic Pontine Glioma. *N. Engl. J. Med.* **2022**, *386*, 2471–2481, doi:10.1056/NEJMoa2202028.
8. Gardner, S.L.; Tarapore, R.S.; Allen, J.; McGovern, S.L.; Zaky, W.; Odia, Y.; Daghistani, D.; Diaz, Z.; Hall, M.D.; Khatib, Z.; et al. Phase I dose escalation and expansion trial of single agent ONC201 in pediatric diffuse midline gliomas following radiotherapy. *Neuro-Oncology Adv.* **2022**, *4*, doi:10.1093/noajnl/vdac143.
9. Majzner, R.G.; Ramakrishna, S.; Yeom, K.W.; Patel, S.; Chinnasamy, H.; Schultz, L.M.; Richards, R.M.; Jiang, L.; Barsan, V.; Mancusi, R.; et al. GD2-CAR T cell therapy for H3K27M-mutated diffuse midline gliomas. *Nature* **2022**, *603*, 934–941, doi:10.1038/s41586-022-04489-4.
10. Panigrahy, A.; Jakacki, R.I.; Pollack, I.F.; Ceschin, R.; Okada, H.; Nelson, M.D.; Kohanbash, G.; Dhall, G.; Bluml, S. Magnetic Resonance Spectroscopy Metabolites as Biomarkers of Disease Status in Pediatric Diffuse Intrinsic Pontine Gliomas (DIPG) Treated with Glioma-Associated Antigen Peptide Vaccines. *Cancers (Basel).* **2022**, *14*, 5995, doi:10.3390/cancers14235995.
11. Mount, C.W.; Majzner, R.G.; Sundaresh, S.; Arnold, E.P.; Kadapakkam, M.; Haile, S.; Labanieh, L.; Hulleman, E.; Woo, P.J.; Rietberg, S.P.; et al. Potent antitumor efficacy of anti-GD2 CAR T cells in H3-K27M+ diffuse midline gliomas. *Nat. Med.* **2018**, *24*, 572–579, doi:10.1038/s41591-018-0006-x.
12. Wei, L.; He, F.; Zhang, W.; Chen, W.; Yu, B. Bioinformatics analysis of microarray data to reveal the pathogenesis of diffuse intrinsic pontine glioma. *Biol. Res.* **2018**, *51*, doi:10.1186/s40659-018-0175-6.

13. Bellat, V.; Alcaina, Y.; Tung, C.H.; Ting, R.; Michel, A.O.; Souweidane, M.; Law, B. A combined approach of convection-enhanced delivery of peptide nanofiber reservoir to prolong local DM1 retention for diffuse intrinsic pontine glioma treatment. *Neuro. Oncol.* **2020**, *22*, 1495–1504, doi:10.1093/neuonc/noaa101.
14. Grimm, S.A.; Chamberlain, M.C. Brainstem glioma: A review. *Curr. Neurol. Neurosci. Rep.* **2013**, *13*, 346, doi:10.1007/s11910-013-0346-3.
15. Buczkowicz, P.; Hawkins, C. Pathology, molecular genetics, and epigenetics of diffuse intrinsic pontine glioma. *Front. Oncol.* **2015**, *5*, 1–9, doi:10.3389/fonc.2015.00147.
16. Vanan, M.I.; Eisenstat, D.D. DIPG in Children – What Can We Learn from the Past? *Front. Oncol.* **2015**, *5*, doi:10.3389/fonc.2015.00237.
17. González, O.E.; Casas, C.; Bermúdez, Y.M. State of the art: pediatric brain stem gliomas. *Rev. Colomb. Cancerol.* **2017**, *21*, 202–211, doi:10.1016/j.rccan.2016.08.002.
18. Gwak, H.S.; Park, H.J. Developing chemotherapy for diffuse pontine intrinsic gliomas (DIPG). *Crit. Rev. Oncol. Hematol.* 2017, *120*, 111–119.
19. Johung, T.B.; Monje, M. Diffuse Intrinsic Pontine Glioma: New Pathophysiological Insights and Emerging Therapeutic Targets. *Curr. Neuropharmacol.* **2017**, *15*, 88–97, doi:10.2174/1570159X14666160509123229.
20. Lapin, D.H.; Tsoli, M.; Ziegler, D.S. Genomic Insights into Diffuse Intrinsic Pontine Glioma. *Front. Oncol.* **2017**, *7*, doi:10.3389/fonc.2017.00057.
21. Hoffman, L.M.; van Zanten, S.E.M.V.; Colditz, N.; Baugh, J.; Chaney, B.; Hoffmann, M.; Lane, A.; Fuller, C.; Miles, L.; Hawkins, C.; et al. Clinical, Radiologic, Pathologic, and Molecular Characteristics of Long-Term Survivors of Diffuse Intrinsic Pontine Glioma (DIPG): A Collaborative Report From the International and European Society for Pediatric Oncology DIPG Registries. *J. Clin. Oncol.* **2018**, *36*, 1963–1972, doi:10.1200/JCO.2017.75.9308.
22. Pollack, I.F.; Agnihotri, S.; Broniscer, A. Childhood brain tumors: Current management, biological insights, and future directions. *J. Neurosurg. Pediatr.* 2019, *23*, 261–273.
23. Vitanza, N.A.; Monje, M. Diffuse Intrinsic Pontine Glioma: From Diagnosis to Next-Generation Clinical Trials. *Curr. Treat. Options Neurol.* **2019**, *21*, 37, doi:10.1007/s11940-019-0577-y.
24. Park, J.; Yea, J.W.; Park, J.W. Hypofractionated radiotherapy versus conventional radiotherapy for diffuse intrinsic pontine glioma. *Medicine (Baltimore).* **2020**, *99*, e22721, doi:10.1097/MD.0000000000022721.
25. Argersinger, D.P.; Rivas, S.R.; Shah, A.H.; Jackson, S.; Heiss, J.D. New developments in the pathogenesis, therapeutic targeting, and treatment of h3k27m-mutant diffuse midline glioma. *Cancers (Basel).* 2021, *13*.
26. Hauser, P. Classification and Treatment of Pediatric Gliomas in the Molecular Era. *Children* **2021**, *8*, 739, doi:10.3390/children8090739.
27. Louis, D.N.; Perry, A.; Wesseling, P.; Brat, D.J.; Cree, I.A.; Figarella-branger, D.; Hawkins, C.; Ng, H.K.; Pfister, S.M.; Reifenberger, G.; et al. The 2021 WHO Classification of Tumors of the Central Nervous System: a summary. *Neuro. Oncol.* **2021**, *23*, 1231–1251, doi:10.1093/neuonc/noab106.
28. Damodharan, S.; Lara-Velazquez, M.; Williamsen, B.C.; Helgager, J.; Dey, M. Diffuse Intrinsic Pontine Glioma: Molecular Landscape, Evolving Treatment Strategies and Emerging Clinical Trials. *J. Pers. Med.* 2022, *12*.
29. Liu, G.; Qiu, Y.; Zhang, P.; Chen, Z.; Chen, S.; Huang, W.; Wang, B.; Yu, X.; Guo, D. Immunogenic Cell Death Enhances Immunotherapy of Diffuse Intrinsic Pontine Glioma: From Preclinical to Clinical Studies. *Pharmaceutics* **2022**, *14*, 1762, doi:10.3390/pharmaceutics14091762.
